# Research on *Babesia*: A bibliometric assessment of a neglected tick-borne parasite

**DOI:** 10.12688/f1000research.17581.2

**Published:** 2019-07-18

**Authors:** Alfonso J. Rodriguez-Morales, D. Katterine Bonilla-Aldana, Juan Pablo Escalera-Antezana, Lucia Elena Alvarado-Arnez

**Affiliations:** 1 Public Health and Infection Research and Incubator Group, Faculty of Health Sciences, Universidad Tecnológica de Pereira, Pereira, Risaralda, 660001, Colombia; 2 School of Medicine, Universidad Franz Tamayo/UNIFRANZ, Cochabamba, 4780, Bolivia; 3 Grupo de Investigación en Ciencias Agropecuarias, Fundación Universitaria Autónoma de las Américas, Pereira, Risaralda, 660003, Colombia; 4 Tongji Hospital & Medical College, Huazhong University of Science & Technology, Wuhan, Hubei, 1037, China

**Keywords:** Babesia, tick-borne disease, epidemiology, public health, bibliometric

## Abstract

Given the emergence and reemergence of tick-borne diseases, here we assessed the publishing patterns of research focused on *Babesia*. We also discuss the implications for the articles published in the last decade, and how more clinical and epidemiological information concerning *Babesia* is still required. The findings of this article would be useful to define research priorities about *Babesia* and diagnose the important of scientific production on this pathogen.

## Introduction

Babesiosis is a zoonotic disease with a global distribution; it is mainly transmitted by ticks from different genera (e.g. *Rhipicephalus* spp *.*, *Dermacentor* spp *.,* and *Ixodes* spp.) and diverse species ^1^. It is caused by infection of the erythrocytes of mammals by *Babesia* species, which are Apicomplexa protozoa of the suborder Piroplasmmiidea and the family Babesiidae ^2^. The vector role of ticks for these parasites was discovered by Smith and Kilbourne in 1893, who were the first to demonstrate its transmission ^3^. The first human case was described by Skaraballo and occurred in 1957 in Zagreb, Croatia ^4^. As a zoonotic disease, animal reservoirs and their distribution contribute, as the presence of vectors, in the maintenance of the transmission cycle and the risk of transmission to humans.

Human babesiosis is not under surveillance and notification in most countries, including those with autochthonous incidence vector-borne diseases. However, studies show that their vectors are widely distributed in tropical and subtropical areas ^3^. Research is fundamental to better understanding this disease. The relevance of bibliometric evaluations on emerging and reemerging disease has been previously described ^5– 7^ as they can contribute in the understanding on how the global scientific and health communities respond to outbreaks ^8^. Herein, our objective was to use bibliometric approaches to analyze *Babesia* research.

## Methods

A bibliometric evaluation was performed focusing on *Babesia* scientific bibliography. Six main databases were used for retrieving information: Science Citation Index Expanded (SCI-E – Web of Knowledge), Scopus, Medline, LILACS, SciELO and Google Scholar.

For the search pipeline we used the following combination of keywords (MeSH, Medical Subject Headings): “Babesia” AND “Latin America”, “ *Babesia*” AND “Argentina”, “ *Babesia*” AND “Colombia”, and this strategy was maintained including the name of each country as a keyword. We searched for the 233 countries of the UN list. Also, “Babesiosis” was used as a substitute of *Babesia* to increase the number of results. Regarding the type of publications, we decided to include original papers, review articles, case reports and editorials, which were further stratified according to publication year and the name and institution to which the main author was affiliated at the time of publishing. This analysis included results obtained up to December 1, 2018.

Data summaries for quantitative variables (number of articles, articles per country, articles per year or periods, citations and H index) were expressed as means and interquartile ranges (IQRs), and for qualitative variables, proportions are reported.

## Results

**Figure 1.**
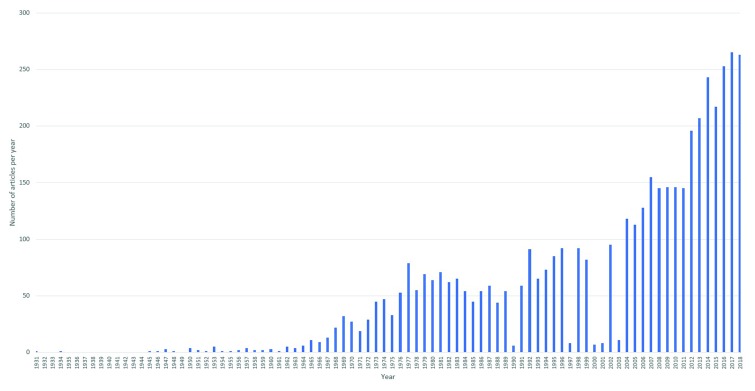
Research trends quantified by the number of published articles on *Babesia* from 1931 to 2018, Medline. In the case of Scopus, the median number of articles published each year as of 1970 was only one (IQR: 0-3), from 1970 until 1995 this number increased to 64 (IQR: 56-73) and from 1996 until 2018 was 188 (IQR: 115–271) ( Figure 2). At Scopus, 134 countries contributed a minimum of one paper over the study period. For SCI-E, the annual median number of articles reported from 1996 until 2018 was of 99 (IQR: 96-103) ( Figure 3), with at least one article published from 129 countries during the study period.

Overall, 78,137 *Babesia*-associated items resulted from the initial screening of publications. From Google Scholar 62,100 articles (25% USA, 24.9% South Africa, 18.5% Japan) were recovered, followed by Scopus, with 6,272 articles (25.4% from USA, 8.5% Japan, 7.2% UK), and Medline with 5.045 articles (13.7% USA, 10.1% Japan and 5.2% China) ( Table 1). From Web of Science, 4,330 publications were retrieved (28.06% from USA, 11.4% Japan and 7.37% Brazil), followed by LILACS with 202 articles (29.2% Brazil, 2.4% Mexico, 1.9% USA) and SciELO with 188 articles (26.6% Brazil, 3.1% Mexico) ( Table 1). Considering the Medline database, the number of research articles on *Babesia* increased above 100 publications per year only after 2004 ( Figure 1).

**Table 1.** The 20 countries with the highest number of scientific articles on *Babesia* research that are available in Web of Science, Scopus and Medline.

Rank	Country	Number of articles	Database with highest number of articles	Population in 2018	Number of articles per 10 million inhabitants
1	United States of America	1594	Scopus	327,096,265	4.87
2	Japan	536	Scopus	127,202,192	4.21
3	United Kingdom	456	Scopus	67,141,684	6.79
4	Australia	424	Scopus	24,898,152	17.03
5	Germany	324	Scopus	83,124,418	3.90
6	Brazil	319	Web of Science	209,469,323	1.52
7	China	284	Web of Science	1,427,647,786	0.20
8	France	256	Scopus	64,990,511	3.94
9	South Africa	254	Web of Science	57,792,518	4.40
10	India	195	Scopus	1,352,642,280	0.14
11	Poland	189	Web of Science	37,921,592	4.98
11	Spain	178	Scopus	46,692,858	3.81
12	Argentina	178	Medline	44,361,150	4.01
13	Italy	172	Scopus	60,627,291	2.84
14	Netherlands	136	Scopus	17,059,560	7.97
15	Turkey	119	Web of Science	82,340,088	1.45
16	Mexico	116	Medline	126,190,788	0.92
17	Switzerland	101	Scopus	8,525,611	11.85
18	Kenya	98	Scopus	51,392,565	1.91
19	Israel	93	Scopus	8,381,516	11.10
20	Egypt	82	Web of Science	98,423,598	0.83

**Figure 2.**
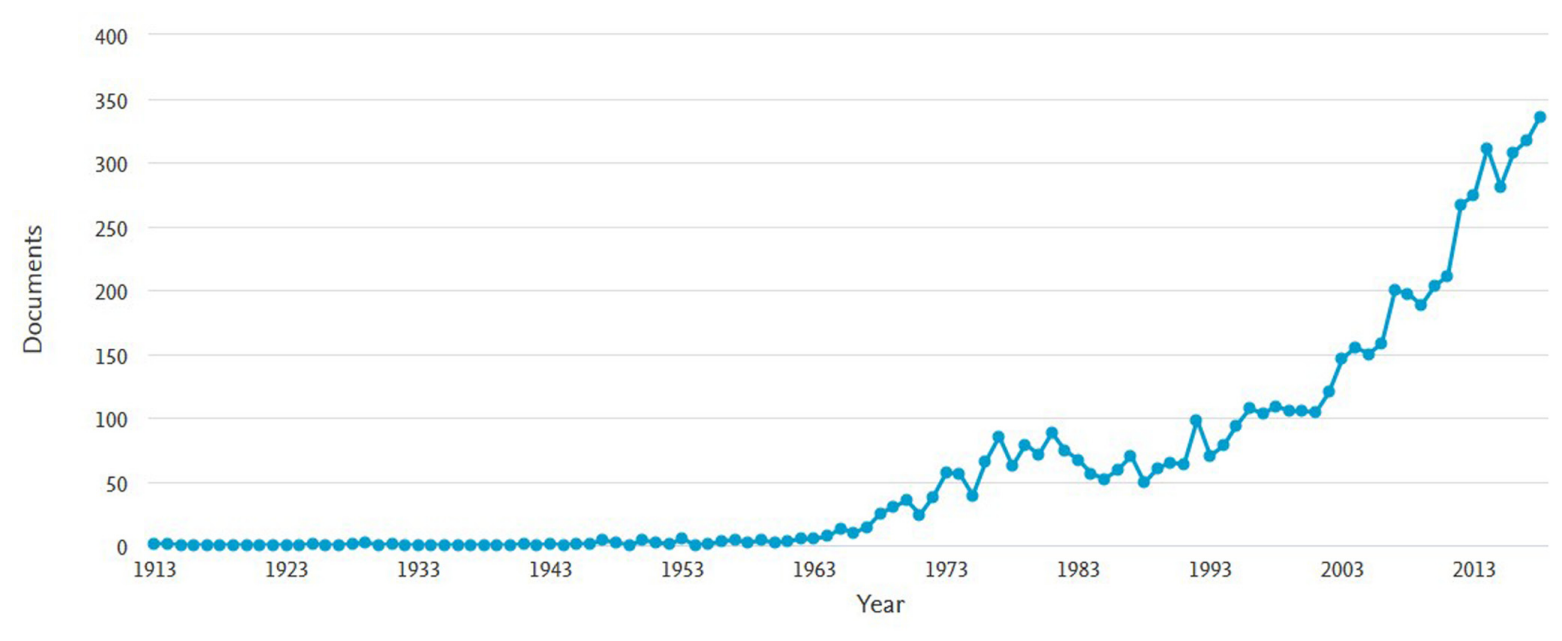
Research trends on *Babesia* from 1931 to 2018, Scopus.

**Figure 3.**
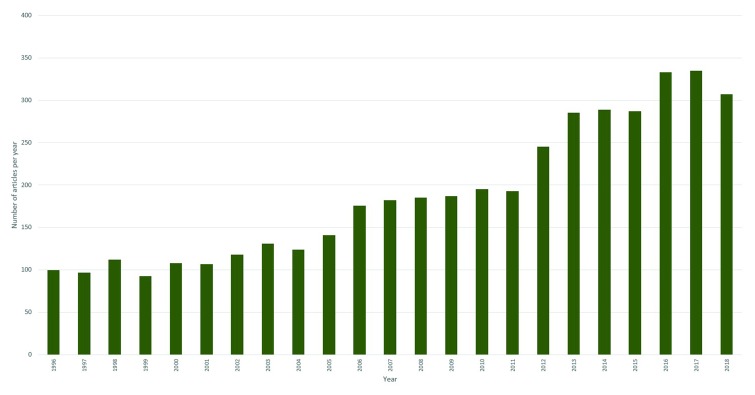
Research trends on *Babesia* from 1996 to 2018, Web of Science.

**Figure 4.**
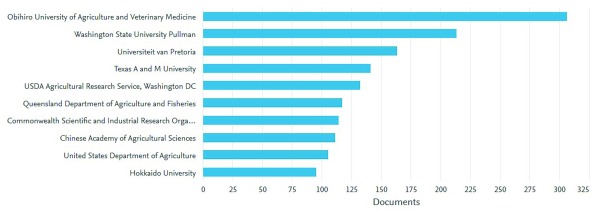
Top research institutions that published scientific literature on *Babesia*, Scopus.

**Figure 5.**
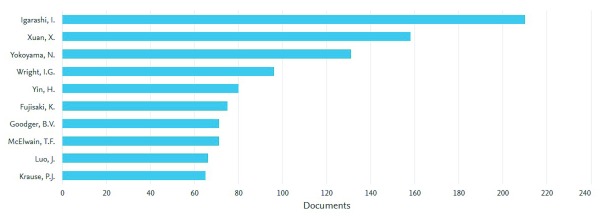
Top research authors that published scientific literature on *Babesia*, Scopus.

**Figure 6.**
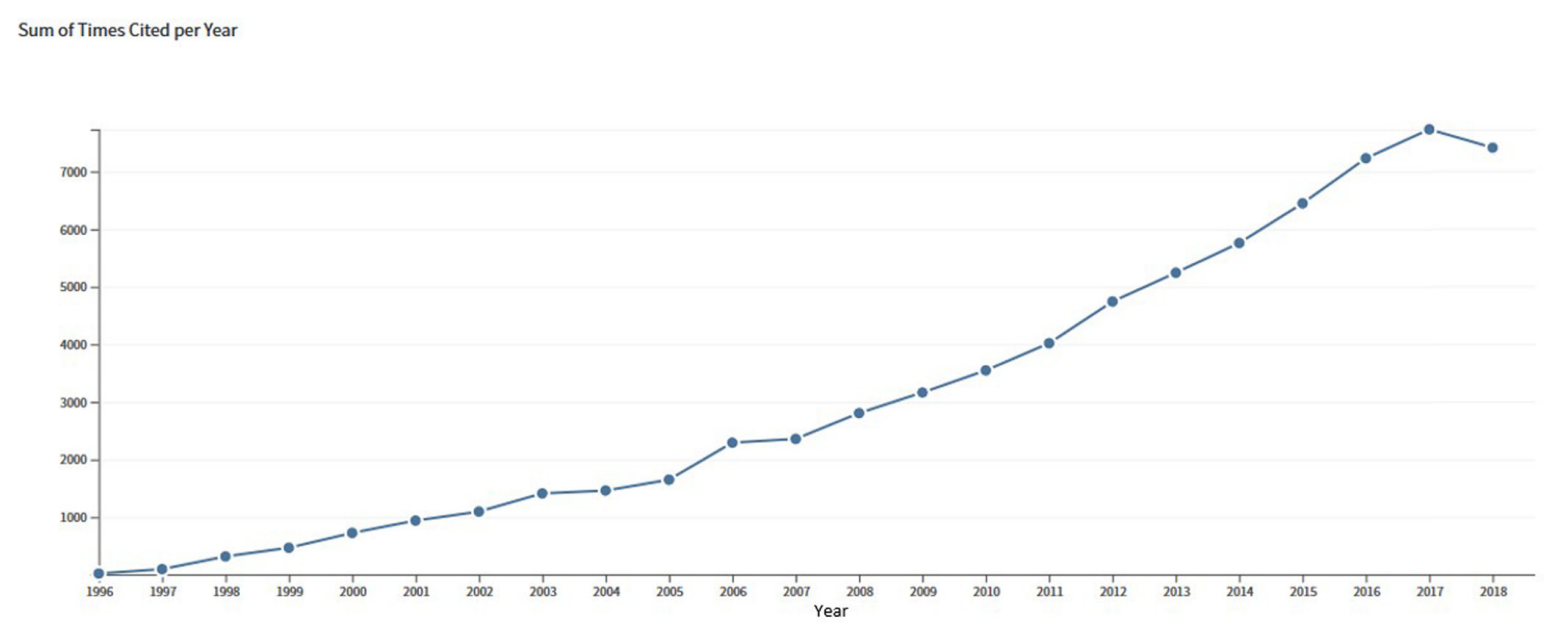
Citation trends on *Babesia* from 1931 to 2018, Web of Science.

“Obihiro University” in Hokkaido, Japan, was the institution with the most productive research in Scopus, and “Igarashi, I” was the author with the largest record in *Babesia* research, with 210 articles ( Figure 4 and Figure 5). At Web of Science, the H index for the topic is 88, with 70,950 citations, reaching 7,734 citations in 2017 ( Figure 6).

**Figure 7.**
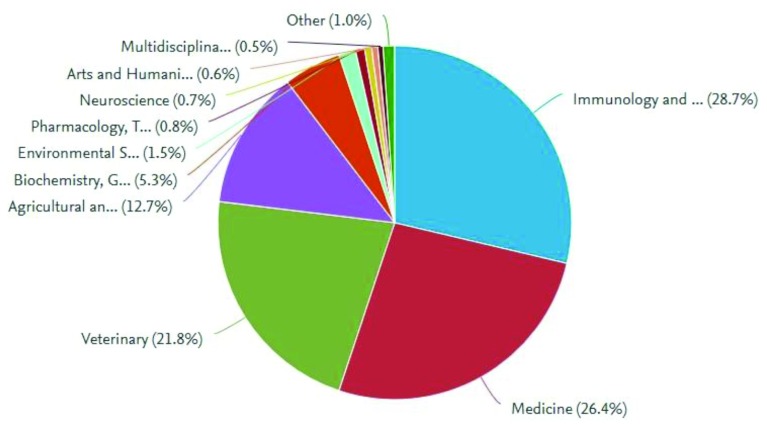
Documents by subject area in Scopus.

**Figure 8.**
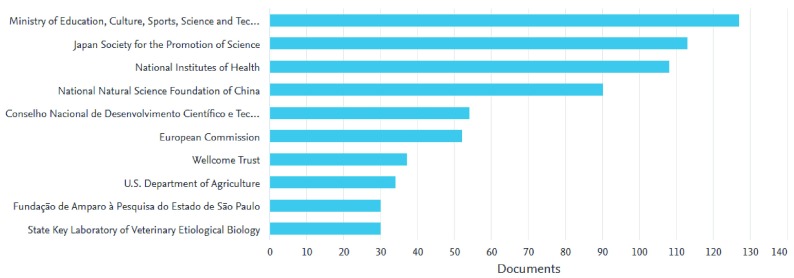
Documents by funding sponsor in Scopus.

Analyzing by areas of research according to Scopus, we found that most of the studies belong to the area of immunology and microbiology (28.7%), followed by medicine (26.4%) and veterinary (21.8%) ( Figure 7). Also in Scopus, by revising the funding sponsors for the published research on *Babesia* ( Figure 8), we found that the Ministry of Education, Culture, Sports, Science, and Technology from Japan, is the main funder (127, 23.7% of Japanese studies), followed by the Japan Society for the Promotion of Science (113, 21.1%) and the National Institutes of Health (108, 6.8%), amongst other funding institutions ( Figure 8).

**Figure 9.**
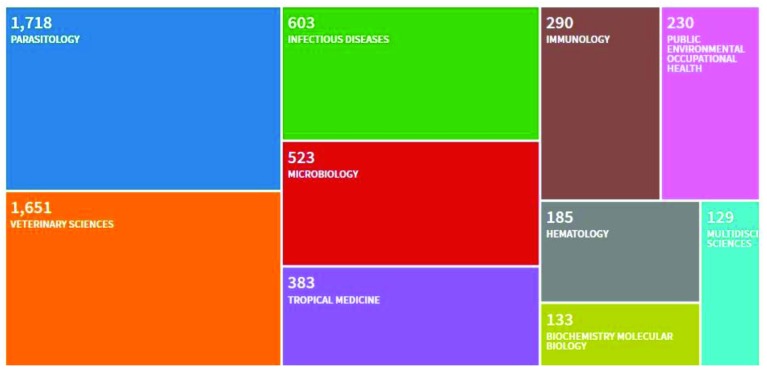
Documents by subject area in Web of Science.

**Figure 10.**
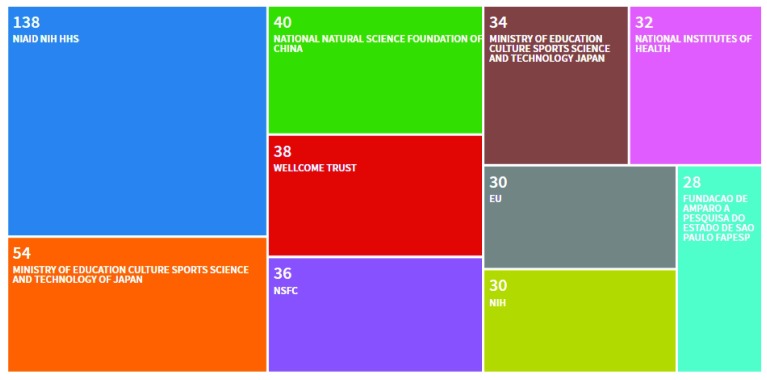
Documents by funding sponsor in Web of Science.

At Web of Science, we found that the areas with more importance for research in Babesia were parasitology (39.2%), veterinary sciences (37.7%), and infectious diseases (13.8%), among others ( Figure 9). Consistent with Scopus, at Web of Science, the National Institutes of Health of USA (138, 8.7%), and the Ministry of Education, Culture, Sports, Science, and Technology of Japan (54, 10.1% of Japanese studies), were the main funders ( Figure 10).

**Figure 11.**
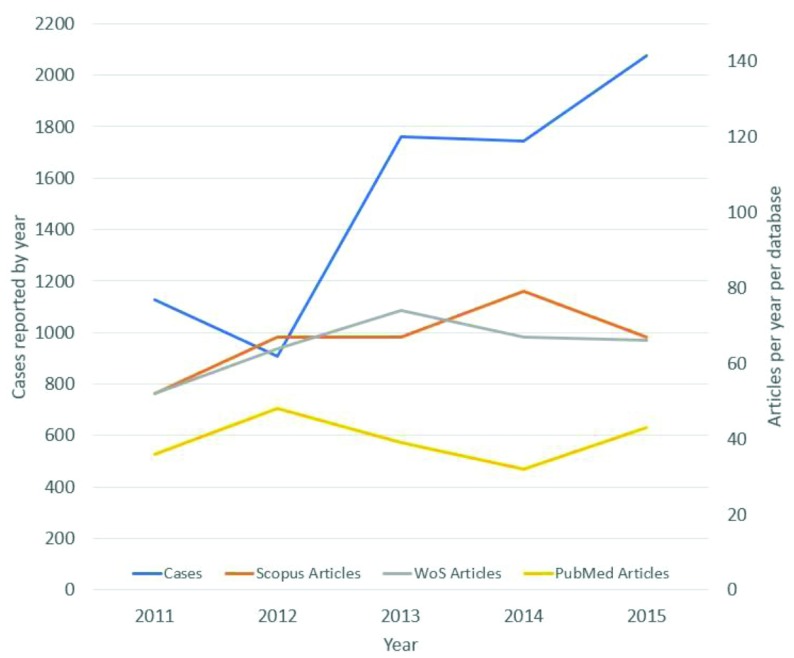
Trends in the number of cases of human babesiosis reported in USA and the number of published articles on babesiosis at Scopus, Web of Science (Wos) and PubMed, 2011–2015.

**Figure 12.**
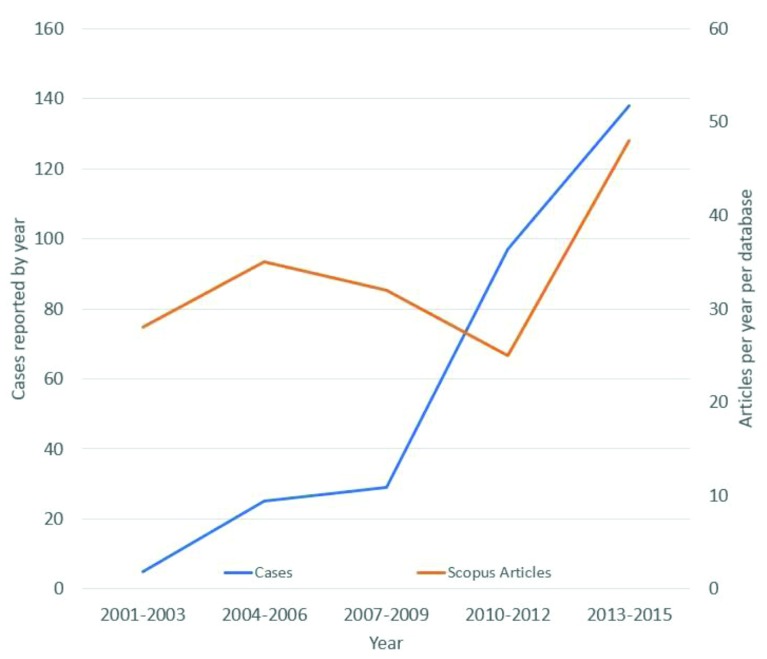
Trends in the number of cases of human babesiosis reported in Wisconsin, USA, and the number of published articles on babesiosis at Scopus, Web of Science (Wos) and PubMed, 2001–2015 from Wisconsin, USA. The raw data generated in this study is available on OSF ^9^.

The scientific production in USA during 2011-2015 was apparently not influenced significantly by the increasing number of babebiosis cases that were reported by surveillance ( Figure 11). However, in Wisconsin, probably the sustained increased observed by the surveillance since 2001, led to an increase in babesiosis research after 2010 ( Figure 12).

## Discussion

The results presented here show that the USA and Japan have primary roles in *Babesia* research, with USA leading the scientific production with nearly quarter of the published articles, followed by Japan and the UK ( Table 1). However, when we calculated the number of articles per million of inhabitants, we found that Australia publish 3.49 more times than USA (and 4.04 times than Japan), followed by Switzerland, Israel, Netherlands, UK and Poland. Certainly, in USA, tickborne disease occurrence is frequent especially in certain areas and months over the year. Tickborne diseases such as babesiosis are commonly reported in Northeastern states as well in the upper Midwest, often with higher incidence in summer. In addition, blood transfusions is still a matter of concern, even in the USA ^10– 13^. In countries in Asia, such as Japan, human babesiosis was not reported until fairly recently (1999), when a symptomatic case was describe in Kobe City, Hyogo Prefecture, Japan ^14, 15^; however, since then research has significantly increased in this country. Authors from UK have collaborated with research with others from endemic countries. However, in 2006 and 2016, two cases of autochthonous canine babesiosis were reported in the UK. Since November 2015, there have been at least three more cases of canine babesiosis in untraveled dogs from Essex, all were confirmed *B. canis* infections by PCR. *Dermacentor reticulatus* ticks were found on the dogs ^16^. The number of articles published by USA and Japan comes as a result from the considerable funding, reflected in the publications supported by their respective agencies. In addition, in USA, babesiosis is a notifiable disease since 2011 (CDC) and most human cases have been reported. Of interest the strong research activity of institutions and countries as Japan and UK, in which *Babesia* represents a new emerging problem in both animals and humans. The findings highlight the increased research activity on this neglected zoonosis, considered of growing importance in several countries and the need of further studies addressed to preventive and therapeutic aspects.

One of the relevant aspects surrounding babesiosis is that there are not yet licensed human prophylactic vaccines, and treatment alternatives remain limited. Two commonly used antimicrobial regimes are highly effective: the combination of atovaquone and azithromycin and the combination of clindamycin and quinine ^17^. Thus, more preventive measures are needed to reduce the risk of infection from ticks and wild and domestic reservoirs (e.g. rats).

The vision of zoonoses should be one. All integrated. Then, having separated human and animal babesiosis, to us, is not rationale today. Babesiosis is one zoonotic disease, no matter the host. The work on babesiosis, including research, should be together between veterinarians and human physicians, working in the interphase that zoonosis, such as babesiosis, provide. One World, One Health. However, as reflected from this bibliometric study, there is a predominance of studies from human medicine compared to veterinary medicine. There is a need for increase of integration with veterinary sciences, given the relevance of babesiosis as a zoonosis.

Bibliometric analyses contribute an objective vision of the scientific activity of a country or a region, in an investigative area. In the particular case of infectious diseases, there are different reports about its utility ^5– 8^, especially in emerging infectious diseases ^18– 20^, being possible to establish and to compare the amount of scientific production in journals, institutions, and authors publishing about a certain issue; this would allow establishment of a plan in terms of scientific policy as well in other matters ^21^. No previous bibliometric studies about babesiosis or *Babesia* have been found in the consulted scientific databases.

It would be ideal to have epidemiological data, such as incidence by active surveillance, but unfortunately such data is not available in most countries, in order to correlate the level of research with the epidemiological relevance of babesiosis. Again, babesiosis is a neglected disease, of importance in several countries, the topic, certainly deserves still more research. Even, in USA, where human babesiosis is now notifiable, only available data is from 2011 to 2015 ^22^, and we retrieved that in order to see if there was a relationship between the number of cases and the number of articles, but this was not apparently influenced, given that during that period, the number of articles from USA did not increased at Scopus, Web of Science and PubMed. However, in Wisconsin, its Department of Health Services, Division of Public Health, in 2001 defines a confirmed case of babesiosis as the occurrence of fever, anemia, or thrombocytopenia in a patient with confirmatory laboratory findings, and its surveillance begun ^23^. Analyzing the number of reported cases from Wisconsin and the number of articles of babesiosis from 2001 to 2015, especially after 2010, epidemiology appears to have influenced an increase in the publications in Scopus.

In conclusion, it is time to translate research findings into effective control of babesiosis. As occurs with other emerging diseases, research leading to vaccinal or effective therapeutic options are of utmost importance. Tick-borne pathogens such as *Babesia* and others with even clearer epidemic potential need to be researched more and to be prioritized with effective interventions to reduce their negative impact.

## Data availability

Raw bibliometric data generated in this study are available on OSF. DOI: https://doi.org/10.17605/OSF.IO/ER9UP ^9^.
