# Supplementary material for: Research on Babesia: A bibliometric assessment of a neglected tick-borne parasite
Source: F1000Res. 2019 Jul 18;7:1987. Originally published 2018 Dec 30. [Version 2] doi: 10.12688/f1000research.17581.2 (PMC6659769; doi:10.12688/f1000research.17581.2)
Supplement: Peer review history

# Peer review: Reviewer response for version 2

**DOI:** 10.5256/f1000research.21848.r51373

**Published:** 2019-07-25

**Authors:** Stalin Vilcarromero

**Affiliations:** 1 Division of Infectious Disease, Department of Medicine, Stony Brook University, New York, NY, USA; 2 Sociedad Científica de Estudiantes de Medicina de la Amazonía Peruana (SOCIEMAP), Facultad de Medicina, Universidad Nacional de la Amazonía Peruana, Iquitos, Peru

In the manuscript entitled "Research on Babesia: A bibliometric assessment of a neglected tick-borne parasite", the authors have included the recommendations made by the reviewers and their quality has improved. This bibliometric review is of interest to future researchers in this zoonosis and shows the need to continue studies in a multidisciplinary manner.

 It is practically ready for indexing, but first, it would be important to answer one question and one minor observation. The first is regarding the comment and graph made considering the state of Wisconsin, considering that there are other states such as Rhode Island, Massachusetts, Connecticut and Maine that show a marked increase in their incidence rates compared to Wisconsin during the period 2011-2014 ^1^. Please clarify, if the same does not happen in the other states.

 The second observation is regarding the writing in some paragraphs of the discussion. For example: "In countries in Asia ..." would be more appropriate: "In Asian countries .." or the following sentence: "Of interest the strong research activity of institutions and countries as Japan and UK, in which Babesia represents a new emerging problem in both animals and humans" the message is not clear. Another example is the paragraph: "The vision of zoonoses should be one. All integrated. Then, having separated human and animal babesiosis, to us, is not rationale today. Babesiosis is one zoonotic disease, no matter the host. The work on babesiosis, including research, should be together between veterinarians and human physicians, working in the interphase that zoonosis, such as babesiosis, provide. One World, One Health." in which redundancies are observed.

I confirm that I have read this submission and believe that I have an appropriate level of expertise to confirm that it is of an acceptable scientific standard.

## References

[1] Surveillance for Babesiosis - United States, 2014 Annual Summary. *U.S. Department of Health and Human Services. [Accessed 25th July 2019]*.2016; Reference source

# Peer review: Reviewer response for version 1

**DOI:** 10.5256/f1000research.19225.r43816

**Published:** 2019-02-28

**Authors:** Cristina Casalone

**Affiliations:** 1 Experimental Institute for Zooprophylaxis in Piedmont, Liguria and Valle D’Aosta, Turin, Italy

The manuscript submitted by Rodríguez Morales et al. represents a bibliometric evaluation on Babesia, in order to contribute to understanding this neglected zoonosis and addressing future research and control strategies. Bibliometric evaluation is an excellent tool to obtain objective information about specific areas of research and support the adoption of strategic decisions. In detail, this study provides and summaries data on the research activity on Babesia worldwide. It shows that the main country involved in the research on Babesia is USA, where babesiosis is a notifiable disease since 2011 (CDC) and most human cases have been reported. Of interest the strong research activity of institutions and countries as Japan and UK, in which Babesia represents a new emerging problem both in animals and humans. This study highlights the increased research activity on this neglected zoonosis, considered of growing importance in several countries and the need of further studies addressed to preventive and therapeutic aspects.

 The manuscript, proposed as a research note, is well structured, the statistical analysis and its interpretation is sufficient, results and discussion appropriate. To fulfil *F1000Research* criteria (“Research note … can be reported with one or two illustrations (figures/tables)”), authors could reduce the number of figures/graphs.

 I suggest the following minor revisions in the text:

In the Introduction:

- I suggest to add *Ixodes *spp as tick genera involved in the transmission of Babesia to humans. Indeed in USA most reported human cases are attributed to *B. microti* transmitted to people by *Ixodes scapularis. *Moreover, most European human cases are caused by *B. divergens* and *B. venatorum*, primarly transmitted by *Ixodes ricinus* ^1^.
- The authors should specify that the first case described in Croatia in 1957 by Skaraballo, refers to a “human” case.
- Moreover I suggest adding a sentence regarding the role of animal reservoirs and their distribution that contributes (as the presence of vectors) in the maintenance of the transmission cycle.

 

In the Methods:

 You could clarify which and/or how many countries have been used as keyword for the search pipeline

 Figure 6: In the caption: the citation trends is from Web of Science (as reported in the results), not Scopus.

I confirm that I have read this submission and believe that I have an appropriate level of expertise to confirm that it is of an acceptable scientific standard.

## References

[1] : Zoonotic babesiosis: overview of the disease and novel aspects of pathogen identity. *Ticks Tick Borne Dis*.2010;1(1) : 10.1016/j.ttbdis.2009.11.003 3-10 10.1016/j.ttbdis.2009.11.003 21771506

# Response

**Published:** 2019-07-07

**Authors:** Alfonso Rodriguez-Morales

**Affiliations:** Universidad Tecnológica de Pereira, Colombia

Dear Dr. Casalone

Thanks for your valuable comments. Regards them, we would like to comment and discuss, in the context of the submission a new revised version (version 2).

**The manuscript submitted by Rodríguez Morales et al. represents a bibliometric evaluation on Babesia, in order to contribute to understanding this neglected zoonosis and addressing future research and control strategies. Bibliometric evaluation is an excellent tool to obtain objective information about specific areas of research and support the adoption of strategic decisions. In detail, this study provides and summaries data on the research activity on Babesia worldwide. It shows that the main country involved in the research on Babesia is USA, where babesiosis is a notifiable disease since 2011 (CDC) and most human cases have been reported. Of interest the strong research activity of institutions and countries as Japan and UK, in which Babesia represents a new emerging problem both in animals and humans. This study highlights the increased research activity on this neglected zoonosis, considered of growing importance in several countries and the need of further studies addressed to preventive and therapeutic aspects.**

We are thankful for your comments. In this revised version, we have included more information such as, an adjustment per capita of the number of publications per million population of the countries (Table 1). In the case of USA, where data from 2011 to 2015 was available, we analyzed and compared the number of reported cases in that period with the number of publications at Scopus, WoS and PubMed during the same time.

**The manuscript, proposed as a research note, is well structured, the statistical analysis and its interpretation is sufficient, results and discussion appropriate. To fulfil F1000Research criteria (“Research note … can be reported with one or two illustrations (figures/tables)”), authors could reduce the number of figures/graphs.**

Thank you for the comments. Per request from the other reviewers, we actually have extended on analyses, data and figures in order to respond to their inquiries.

**I suggest the following minor revisions in the text:**

**In the Introduction:**

**I suggest to add *Ixodes spp* as tick genera involved in the transmission of Babesia to humans.**

Done. Included (Second line in Introduction section).

**Indeed, in USA most reported human cases are attributed to *B. microti* transmitted to people by Ixodes scapularis. Moreover, most European human cases are caused by *B. divergens* and B. *venatorum*, primarly transmitted by *Ixodes ricinus1*.**

Done. Included.

**The authors should specify that the first case described in Croatia in 1957 by Skaraballo, refers to a “human” case.**

Done. Included (Fifth line in Introduction section).

**Moreover, I suggest adding a sentence regarding the role of animal reservoirs and their distribution that contributes (as the presence of vectors) in the maintenance of the transmission cycle.**

Done, now included (Final sentence in Introduction section).

**In the Methods:**

**You could clarify which and/or how many countries have been used as keyword for the search pipeline**

Now included (In Methods section after keyword specification).

**Figure 6: In the caption: the citation trends is from Web of Science (as reported in the results), not Scopus.**

Corrected.

# Peer review: Reviewer response for version 1

**DOI:** 10.5256/f1000research.19225.r44953

**Published:** 2019-02-26

**Authors:** Jeremy S. Gray

**Affiliations:** 1 UCD School of Biology & Environmental Science, University College Dublin, Dublin, Ireland

This article attempts to assess the bibliographic status of *Babesia* parasites with the declared objective of identifying research priorities in order to achieve effective prevention and control of babesiosis. The authors have produced publication data from various sources showing trends over the years and also by citation, author, institute and country.

 Unfortunately the article has major deficiencies. The most obvious of these is that the human and animal versions of the disease have been conflated so that the data are more or less meaningless. The economic impact, research priorities and research constraints are very different in veterinary compared with medical babesiosis. In fact it is possible to argue that even the parasites are different, since the vast majority of human cases are caused by a parasite ( *Babesia microti*) that is only distantly related to those prevalent in veterinary babesiosis ( *Babesia* sensu stricto), and there are differences in their biology such as presence or absence of transovarial transmission, sensitivity to antibabesials, availability of in vitro cultures etc.

 Additionally, it is difficult to see the point of counting numbers of articles published by different countries, authors, institutions etc. Cross comparison of these data is invidious since different interests and time scales are involved. Such data may tell the reader something about where there has been sufficient interest for research funding but nothing about the nature of the research, which is necessary to identify areas of neglect. It would have been much more useful to break the data down by topic. For example, how many articles are in the area of pure immunological research, usually involving rodents, how many address therapeutic issues, how many vaccination, how many epidemiology etc. Only then would it be possible to see where the gaps are, particularly in relation to practical measures, particularly if accompanied by analytical comments. The superficial approach of this article certainly does not, especially when there has been no attempt to explain the trends presented in the figures.

 The authors have identified some research areas that require more attention, for example blood transfusion infection in the USA, development of vaccines (presumably molecular), development of new antibabesials, but have not provided the necessary context or evidence for these conclusions.

 Some important issues that the authors seem to have ignored completely, include the development and successful use of live vaccines for cattle babesiosis over a long period of time, the prodigious, but failed efforts, to produce molecular vaccines against cattle babesiosis (which indicates the very great difficulty involved in the development of vaccines for human use), the change in direction and emphasis of babesiosis research in general with the discovery of *B. microti *in the US, and epidemiological issues such as the extension of the geographical range of infections, although briefly touched on in relation to the UK, for reasons that are not clear.

 The references make little mention of established authorities in the topic and there are at least five instances of self-citation. Finally there are many examples of poor sentence construction (e.g. the last part of the last sentence in the abstract, inaccurate statements (e.g. the first sentence in the Introduction and the first sentence in the second paragraph), unnecessary sentences (e.g. the third sentence in the second paragraph) etc. There are more of all these in the Discussion.

 Overall, the impression gained is that the authors have made use of readily available metrics on the internet, to present data that appear to have no useful meaning and have not attempted to analyze the data to achieve their stated objectives.

I confirm that I have read this submission and believe that I have an appropriate level of expertise to state that I do not consider it to be of an acceptable scientific standard, for reasons outlined above.

# Response

**Published:** 2019-07-07

**Authors:** Alfonso Rodriguez-Morales

**Affiliations:** Universidad Tecnológica de Pereira, Colombia

Dear Dr. Gray

Thanks for your comments. We have revised and improved the manuscript. In regard to your observations, we would like to comment and discuss, in the context of the submission a new revised version (version 2).

**This article attempts to assess the bibliographic status of Babesia parasites with the declared objective of identifying research priorities in order to achieve effective prevention and control of babesiosis. The authors have produced publication data from various sources showing trends over the years and also by citation, author, institute and country.**

The objective of this bibliometric study was not to “identifying research priorities in order to achieve effective prevention and control of babesiosis”, which would be impossible from this type of study. What we really declared is “The findings of this article would be useful to define research priorities about Babesia and diagnose the important of scientific production on this pathogen” (final line of the abstract)

**Unfortunately the article has major deficiencies. The most obvious of these is that the human and animal versions of the disease have been conflated so that the data are more or less meaningless. The economic impact, research priorities and research constraints are very different in veterinary compared with medical babesiosis. In fact it is possible to argue that even the parasites are different, since the vast majority of human cases are caused by a parasite ( *Babesia microti*) that is only distantly related to those prevalent in veterinary babesiosis ( *Babesia *sensu stricto), and there are differences in their biology such as presence or absence of transovarial transmission, sensitivity to antibabesials, availability of in vitro cultures etc.**

We thank the reviewer for the observation, although we consider that today, the vision of zoonoses should be an integrated one. Then, having separated human and animal babesiosis, to us, is not rationale. Babesiosis is one zoonotic disease, no matter the host. The work on babesiosis should be together between veterinarians and human physicians, working in the interphase that zoonoses, such as babesiosis, provide. One World, One Health.

**Additionally, it is difficult to see the point of counting numbers of articles published by different countries, authors, institutions etc. Cross comparison of these data is invidious since different interests and time scales are involved. Such data may tell the reader something about where there has been sufficient interest for research funding but nothing about the nature of the research, which is necessary to identify areas of neglect. It would have been much more useful to break the data down by topic. For example, how many articles are in the area of pure immunological research, usually involving rodents, how many address therapeutic issues, how many vaccination, how many epidemiology etc. Only then would it be possible to see where the gaps are, particularly in relation to practical measures, particularly if accompanied by analytical comments. The superficial approach of this article certainly does not, especially when there has been no attempt to explain the trends presented in the figures.**

Bibliometric studies quantify and compare the scientific output on specific and general topics. That is the idea and the design, that has been a base consensus in this type of studies. Nevertheless, we have included additional information in regards the areas of research related to Babesia as well as the funding sponsors, from both Scopus and Web of Sciences.

**The authors have identified some research areas that require more attention, for example blood transfusion infection in the USA, development of vaccines (presumably molecular), development of new antibabesials, but have not provided the necessary context or evidence for these conclusions.**

That would be more related to other kind of studies. If required, a specific study (e.g. review) about that should be designed, to address those specific topics.

**Some important issues that the authors seem to have ignored completely, include the development and successful use of live vaccines for cattle babesiosis over a long period of time, the prodigious, but failed efforts, to produce molecular vaccines against cattle babesiosis (which indicates the very great difficulty involved in the development of vaccines for human use), the change in direction and emphasis of babesiosis research in general with the discovery of *B. microti* in the US, and epidemiological issues such as the extension of the geographical range of infections, although briefly touched on in relation to the UK, for reasons that are not clear.**

We agree with the reviewer that such points would be of interest. But our study characterizes as a bibliometric research note, and  not a systematic review, or a bibliometric study about vaccines. Therefore, that was not an objective of our study.

**The references make little mention of established authorities in the topic and there are at least five instances of self-citation. Finally there are many examples of poor sentence construction (e.g. the last part of the last sentence in the abstract, inaccurate statements (e.g. the first sentence in the Introduction and the first sentence in the second paragraph), unnecessary sentences (e.g. the third sentence in the second paragraph) etc. There are more of all these in the Discussion.**

Thank you very much for the observation. In this revised version, we attended several of such issues in order to improve the manuscript.

**Overall, the impression gained is that the authors have made use of readily available metrics on the internet, to present data that appear to have no useful meaning and have not attempted to analyze the data to achieve their stated objectives.**

As previously indicated, in this revised version the manuscript has been improved. Although we would like to clarify that: i) the metrics presented in this research note were not available on the internet and ii) the databases that were evaluated are significant sources for bibliometric studies, to whom (Scopus and SCI-E Web of Knowledge) the Universidad Tecnológica de Pereira, in Colombia has valid subscriptions.

# Peer review: Reviewer response for version 1

**DOI:** 10.5256/f1000research.19225.r42304

**Published:** 2019-02-08

**Authors:** Stalin Vilcarromero

**Affiliations:** 1 Division of Infectious Disease, Department of Medicine, Stony Brook University, New York, NY, USA; 2 Sociedad Científica de Estudiantes de Medicina de la Amazonía Peruana (SOCIEMAP), Facultad de Medicina, Universidad Nacional de la Amazonía Peruana, Iquitos, Peru

In the manuscript entitled “Research on Babesia: A bibliometric assessment of a neglected tick-borne parasite” the authors try to evaluate the previous bibliometric research regarding babesiosis in the world. It has recognized the value of this type of study because it helps to identify the importance of a country, institution or researcher in solving problems based on scientific evidence. Most of them describe bibliometrics in their papers considering bibliometric variables such as number of citations, author participation in research production, author and co-authorship analysis with VOSviewer ^1^, the H-index, geographical distribution of that disease by countries, the amount and intensity of their international collaboration, analysis of that research based on the frequency of the words used in the title of the articles ^2^, number of publications with intra-country collaboration, number of publications with inter-country collaboration ^3^^,^^4^ etc, and usually, the literature was retrieved using only one database (Scopus, Medline, etc) which may give the advantage to let them analyze this in depth.

 However, when the number of publications is normalized by population ^1^, by gross domestic product, and by gross national income per capita ^5^, health expenditure ^6^, scientific collaboration ^7^ or other important variable (epidemiology variables such as prevalence, incidence ^8^, endemic versus non-endemic, etc) it makes more relevant the study. On the other hand, it is known the capacity of databases such Scopus, Medline, Web of Science and Scielo, and the authors may decide to use only one giving more details to the analysis.

 In this case, considering that Babesiosis is a neglected disease, of importance in several countries as it has been described in the manuscript, so this topic deserve still more research, so I consider that this bibliometric analysis would be important for the scientific community. However, it would also be important to normalize the number of publications (including some of those variables mentioned above), include other bibliometric variables such as H-index.

I confirm that I have read this submission and believe that I have an appropriate level of expertise to confirm that it is of an acceptable scientific standard, however I have significant reservations, as outlined above.

## References

[1] : A bibliometric analysis of the global research on biosimilars. *J Pharm Policy Pract*.2018;11: 10.1186/s40545-018-0133-2 6 10.1186/s40545-018-0133-2 29599984PMC5870206

[2] : A decade bibliometric analysis of global research on leishmaniasis in Web of Science database. *Ann Med Surg (Lond)*.2018;26: 10.1016/j.amsu.2017.12.014 30-37 10.1016/j.amsu.2017.12.014 29387384PMC5771967

[3] : Public, environmental, and occupational health research activity in Arab countries: bibliometric, citation, and collaboration analysis. *Arch Public Health*.2015;73(1) : 10.1186/2049-3258-73-1 1 10.1186/2049-3258-73-1 25671116PMC4322552

[4] : A bibliometric analysis of global research on toxoplasmosis in the Web of Science. *Vet World*.2018;11(10) : 10.14202/vetworld.2018.1409-1415 1409-1415 10.14202/vetworld.2018.1409-1415 30532494PMC6247882

[5] : A bibliometric analysis by geographic area of published research in several biomedical fields, 1995-2003. *CMAJ*.2006;175(11) : 10.1503/cmaj.060361 1389-90 10.1503/cmaj.060361 17116906PMC1635765

[6] : Bibliometric analysis of leishmaniasis research in Medline (1945-2010). *Parasit Vectors*.2013;6: 10.1186/1756-3305-6-55 55 10.1186/1756-3305-6-55 23497410PMC3602049

[7] : South American collaboration in scientific publications on leishmaniasis: bibliometric analysis in SCOPUS (2000-2011). *Rev Inst Med Trop Sao Paulo*.56(5) :381-90 2522921710.1590/S0036-46652014000500003PMC4172108

[8] : A bibliometric overview of infectious diseases research in European countries (2002-2007). *Eur J Clin Microbiol Infect Dis*.2009;28(6) : 10.1007/s10096-008-0691-3 713-6 10.1007/s10096-008-0691-3 19139934

# Response

**Published:** 2019-07-07

**Authors:** Alfonso Rodriguez-Morales

**Affiliations:** Universidad Tecnológica de Pereira, Colombia

Dear Dr. Vilcarromero

Thanks for your valuable comments. Regards them, we would like to comment and discuss, in the context of the submission a new revised version (version 2).

**In the manuscript entitled “Research on Babesia: A bibliometric assessment of a neglected tick-borne parasite” the authors try to evaluate the previous bibliometric research regarding babesiosis in the world. It has recognized the value of this type of study because it helps to identify the importance of a country, institution or researcher in solving problems based on scientific evidence. Most of them describe bibliometrics in their papers considering bibliometric variables such as number of citations, author participation in research production, author and co-authorship analysis with VOSviewer1, the H-index, geographical distribution of that disease by countries, the amount and intensity of their international collaboration, analysis of that research based on the frequency of the words used in the title of the articles2, number of publications with intra-country collaboration, number of publications with inter-country collaboration3,4 etc, and usually, the literature was retrieved using only one database (Scopus, Medline, etc) which may give the advantage to let them analyze this in depth.**

We performed a bibliometric study; we did not evaluate previous bibliometric research. As until the present date, there are no records of previous published bibliometric study in this subject. Regarding the “method”, there is still no consensus on reporting or performing bibliometric studies. Some studies analyze only one database, but this would lead to certain bias, as e.g. WoS have a limited geographical coverage, compared to Scopus. Or Medline is limited in number of journals of certain biomedical areas. The advantage of having different databases is to have a wider coverage and avoid geographical bias, as we intended to do. Secondly, we have published a Research Note (a brief article), not an Original Article. Then extension is quite different. In reference to variables, we used the most common ones utilized in bibliometric studies.

**However, when the number of publications is normalized by population** **^1^** **, by gross domestic product, and by gross national income per capita** **^5^** **, health expenditure** **^6^** **, scientific collaboration** **^7^** **or other important variable (epidemiology variables such as prevalence, incidence** **^8^** **, endemic versus non-endemic, etc) it makes more relevant the study. On the other hand, it is known the capacity of databases such Scopus, Medline, Web of Science and Scielo, and the authors may decide to use only one giving more details to the analysis.**

Although the proposed relationships would be very relevant, this is a research note for a descriptive bibliometric study. Such analysis would result in an extended analytical bibliometric study for a Full-Length Original Article. Even more, some of the suggested variables are not standardized for many countries nor available for the whole period of years and even more publicly available. For example, as we clearly stated, babesiosis is not under surveillance in most countries. Then, this makes highly unlikely to have epidemiological indicators to make such correlations. However, we included in the Table 1 an adjustment per capita of the number of publications per million population of the countries. In the case of USA, where data from 2011 to 2015 was available, we analyzed and compared the number of reported cases in that period with the number of publications at Scopus, WoS and PubMed during the same time. That data from USA, was published this year (May 2019), and was now cited by us, but not previously available, when we performed the bibliometric study. For more years, and other countries, this is not possible. In addition, we have access to data from Wisconsin, USA, which was included and analyzed (Figure 12).

**In this case, considering that Babesiosis is a neglected disease, of importance in several countries as it has been described in the manuscript, so this topic deserve still more research, so I consider that this bibliometric analysis would be important for the scientific community. However, it would also be important to normalize the number of publications (including some of those variables mentioned above), include other bibliometric variables such as H-index.**

We are grateful to the reviewer in the appreciation that the bibliometric analysis is of relevance for the scientific community. In reference of the mentioned variables, the H index is already included in the manuscript (third paragraph of the results description).
